# A new *Bacillus thuringiensis* protein for Western corn rootworm control

**DOI:** 10.1371/journal.pone.0242791

**Published:** 2020-11-30

**Authors:** Yong Yin, Stanislaw Flasinski, William Moar, David Bowen, Cathy Chay, Jason Milligan, Jean-Louis Kouadio, Aihong Pan, Brent Werner, Karrie Buckman, Jun Zhang, Geoffrey Mueller, Collin Preftakes, Bruce E. Hibbard, Paula Price, James Roberts

**Affiliations:** 1 Bayer Crop Science, Chesterfield, Missouri, United States of America; 2 USDA-ARS, Plant Genetics Research Unit, University of Missouri, Columbia, Missouri, United States of America; University of Tennessee, UNITED STATES

## Abstract

The Western corn rootworm (WCR) *Diabrotica virgifera virgifera* LeConte is one of the most economically important insect pests in North America. Since 2003, transgenic maize expressing WCR-active proteins from *Bacillus thuringiensis* (*Bt*) have been widely adopted as the main approach to controlling WCR in the U.S. However, the emergence of field resistance to the *Bt* proteins in current commercial products has been documented in recent years, highlighting the need to develop additional tools for controlling this devasting pest. Here we report the discovery of Vpb4Da2 (initially assigned as Vip4Da2), a new insecticidal protein highly selective against WCR, through high-throughput genome sequencing of a *Bt* strain sourced from grain dust samples collected in the eastern and central regions of the US. Vpb4Da2 contains a sequence and domain signature distinct from families of other WCR-active proteins. Under field conditions, transgenic maize expressing Vpb4Da2 demonstrates commercial-level (at or below NIS 0.25) root protection against WCR, and reduces WCR beetle emergence by ≥ 97%. Our studies also conclude that Vpb4Da2 controls WCR populations that are resistant to WCR-active transgenic maize expressing Cry3Bb1, Cry34Ab1/Cry35Ab1 (reassigned as Gpp34Ab1/Tpp35Ab1), or DvSnf7 RNA. Based on these findings, Vpb4Da2 represents a valuable new tool for protecting maize against WCR.

## Introduction

Since the beginning of the 20^th^ century, the rod-shaped, aerobic endospore-forming *Bacillus thuringiensis* (*Bt*) has been well known to cause insect diseases as entomopathogens [1]. A vast variety of insecticidal proteins have been identified from *Bt* and have been systematically classified in the *Bacillus thuringiensis* Toxin Nomenclature since 1998 based on their primary sequence homologies [2]. In July 2020, a revised, structure-based nomenclature for *Bt* and other bacteria-derived pesticidal proteins was published to reflect the diverse protein folds from a wider range of bacteria while maintaining relationships with the existing nomenclature [3, 4].

Several families of *Bt* crystal (Cry) proteins, named for being produced as protein crystals during *Bt* sporulation, have been utilized commercially in transgenic crops for insect protection. The Cry1 and Cry2 proteins used against lepidopteran insects and Cry3 proteins against coleopteran pests are typical three-domain Cry proteins, as they contain a conserved structural fold consisting of three individually folded protein domains. They are classified as α-pore forming proteins (α-PFPs) for forming α-helical transmembrane pores on the insect gut epithelium that ultimately lead to target pest mortality [5]. *Bt* proteins from two other Cry families have also been developed as transgenic traits, both categorized as β-pore forming proteins (β-PFPs) as they form β-barrel transmembrane pores. Cry35Ab1 (reassigned as Tpp35Ab1 in the revised nomenclature) from the Toxin_10 β-PFP family acts together with a binary partner Cry34Ab1 (reassigned as Gpp34Ab1) in producing toxicity to Western corn rootworm (WCR) *Diabrotica virgifera virgifera* LeConte [6, 7]. More recently, an engineered variant of Cry51 (reassigned as Mpp51) from the ETX-MTX2 β-PFP family has been developed for expressions in transgenic cotton for protection against *Lygus* and thrips species [8–11].

In addition to Cry proteins, *Bt* strains produce several families of insecticidal proteins, known as Vegetative Insecticidal Proteins (Vip), during the vegetative phase of growth [12]. Among the Vip proteins identified to date, Vip3Aa from the Vip3 family has been widely commercialized in transgenic crops against lepidopteran pests [12]. It has a unique sequence and different structural fold compared to three-domain and other Cry proteins, with evidence suggesting a pore formation-based mechanism of action [13–15].

WCR is one of the most devastating insect pests of maize in North America, which can cause extensive crop damage and consequent economic losses to growers exceeding $1 billion annually if not controlled [16]. One of the key developments since 2003 in the U.S. has been the wide adoption of commercial maize hybrids expressing individual or pyramided *Bt* insecticidal proteins for protection against WCR, which has contributed to increased crop productivity and reduced insecticide use in maize in North America [17, 18]. Currently, commercially available transgenic maize events targeting WCR are based on *Bt* proteins Cry3Bb1, Cry34Ab1/Cry35Ab1, mCry3A, and eCry3.1Ab [7, 19, 20]. In recent years though, the emergence of WCR field resistance to these proteins has been reported [21–24], highlighting the need to develop additional tools for protection against this economically important pest. The recent development of DvSnf7 RNA [25, 26] and a novel protein (IPD072Aa) discovered from *Pseudomonas chlororaphis* [27] are examples of next generation transgenic maize that are able to control WCR populations resistant to *Bt* proteins in current commercial products.

Here we report the discovery of Vpb4Da2 (initially assigned as Vip4Da2), a new *Bt* protein from the Vpb4 (formally Vip4) insecticidal protein family [2–4]. Our studies demonstrate that Vpb4Da2 has highly selective insecticidal activity against WCR, including WCR populations resistant to current commercial WCR-active *Bt* proteins. We conclude that transgenic maize expressing Vpb4Da2 can provide a new tool for the control of WCR.

## Materials and methods

### Discovery, cloning, and bacterial expression of Vpb4Da2

*Bt* strain EG6657 was isolated in 1986 as part of a *Bt* collection sourced from grain dust samples obtained from farmers in the eastern and central regions of the US. As described previously [28, 29], grain dust samples were treated and spread on NYSM agar plates to isolate *Bacillus*-type colonies. *Bt* strains were identified based on visual detection of phase bright spore and crystalline inclusions in sporulating colonies using phase-contrast microscopy. They were further analyzed by a modified Eckhardt agarose gel electrophoresis procedure [30] to determine the array of native plasmids found in each strain. Strains with unique plasmid arrays and crystal morphologies were selected for detailed study.

An EG6657 genomic DNA sequencing library was prepared using Nextera DNA Library Kit (Illumina, San Diego, CA) and sequenced on Illumina HiSeq. A draft genome assembly was generated using CLCBio Genomics Workbench, from which the ORF encoding Vpb4Da2 was identified based on BLAST and Pfam searches for sequence conservation in known insecticidal proteins. PCR Primers were designed based on the first 30 nucleotides of the 5’ end and the last 30 nucleotides of the 3’ end of the coding region to generate a PCR product for cloning. Also included in the design were an additional 16 nucleotides to enable hot fusion cloning [31]. PCR products were generated from genomic DNA using standard PCR conditions with KOD Hot Start DNA polymerase (Novagen, San Diego, CA) and cloned into a *Bt*/*E*. *coli* shuttle vector plasmid. A Vpb4Da2-C-His PCR product was generated from the plasmid above using standard PCR conditions, followed by cloning into an *E*. *coli* expression vector with hot fusion. The resulting plasmid was transformed into *E*. *coli* Rosetta2 (DE3) competent cells for protein expression. Transformed *E*. *coli* cells were grown in auto-induction media at 37°C. The C-terminal His-tagged Vpb4Da2 protein was purified using batch nickel chromatography and buffer-exchanged into 25 mM carbonate buffer (pH 10) and 5mM DTT, estimated to be greater than 90% pure by SDS-PAGE on Gel Doc EZ system (Bio-Rad Laboratories, Hercules, CA), and was quantified by the Bradford assay and stored at -80°C.

### Diet-overlay bioassays on WCR and Northern corn rootworm (NCR, *Diabrotica barberi*)

Diet-overlay bioassays were conducted on WCR and NCR as described previously [26]. Briefly, 20 μl of Vpb4Da2 at 0.5 μg/μl, buffer control, and water control were overlaid onto the diet surface of each well in an 8-well column on 96-well plates, repeated three or four times on different plates. This resulted in a final concentration of Vpb4Da2 of 31.25 μg/cm^2^. Bioassays were conducted with WCR or NCR neonates (<24 hour old), one insect per well, and scored on day 5. Larvae were considered dead if they were immobile when touched with a fine tip paintbrush. Any bioassay was discarded if more than 30% mortality or less than 70% infestation was detected on the water control, or more than 15% contamination was observed. Mortality and stunting were evaluated in a column-wise manner. Mortality for a column was calculated from the number of wells with dead larvae in the column. The stunting score per non-water column was determined using a 0–3 scale based on visual difference in larval size between the column and the water column on the same plate, with a score of 0, 1, 2, or 3 defined as less than a <25%, 25–50%, 50–75%, or >75% difference in size, respectively. Average (±SD) mortality and stunting scores for each non-water treatment were calculated from all the columns per treatment.

### Diet-incorporation bioassays for assessing activity spectrum

Diet-incorporation bioassays were conducted as described previously [32] to test for effects of Vpb4Da2 on multiple insect species from Coleoptera (*Diabrotica undecimpunctata*, *Epilachna varivestis*, *Leptinotarsa decemlineata*), Lepidoptera (*Ostrinia nubilalis*, *Helicoverpa zea*, *Spodoptera frugiperda*), and Hemiptera (*Lygus hesperus*). Treatments included a water control, buffer control, and Vpb4Da2 at 500 μg/g diet. WCR activity was evaluated at 250 μg Vpb4Da2/g diet as a positive control. For each test species, exposures to dietary treatments were initiated concurrently with a target total of 16–36 insects for each treatment. All exposures were initiated as neonates (<30 hr old), and tested organisms were allowed to feed *ad libitum* for six to seven days. Any assay was discarded if mortality ≥ 10% on the water control. Percent survival and average body mass were collected at completion of the assays.

### Generation of Vpb4Da2 transgenic maize

Vpb4Da2 codons were redesigned for optimal expression in monocots with a targeted GC content of 57% (GenBank accession number MT611519). Binary vectors containing candidate cassettes to drive the expression of Vpb4Da2 were cloned with an additional CP4-EPSPS selection cassette providing resistance to glyphosate. They were subsequently transformed to the maize inbred line LH244 using *Agrobacterium*-mediated transformation. Single-copy events were selected based on molecular assays and grown to produce hybrid F1 seeds by transferring pollen from transgenic plants to donor ears in the 93IDI3 inbred line for further studies. The selection of expression cassettes and Vpb4Da2 transgenic maize lines are described in S1 Method and S1 Table.

### Growth chamber whole-plant root protection assay

Hybrid seeds from eleven Vpb4Da2 transgenic maize lines, commercial product SmartStax® expressing both Cry3Bb1 and Cry34Ab1/Cry35Ab1, and wild-type maize were individually germinated and grown in 2.36 L containers (U.S. Plastics, Lima, OH) containing Berger BM6 soil in a growth chamber at 25°C day/21°C night, 16:8 h light/dark, 50% relative humidity (RH) and 650 lum. Plant identity was confirmed through tissue sampling and ELISA. Eggs from a non-diapausing susceptible lab WCR colony (Waterman, IL) were incubated at 25°C, 60% RH in total darkness for 13 days immediately prior to hatch. At approximately V4, six plants for each maize line were infested with approximately 2,000 WCR eggs per plant. 24 days after infestation, roots were removed from pots and evaluated for root damage using the 0–3 Node-Injury Scale (NIS) [33]. Mean (±SEM) NIS scores were calculated from NIS ratings of all six plants for each maize line.

A whole-plant assay was conducted using NCR eggs from a non-diapausing lab colony (Crop Characteristics, Inc., Farmington, NM). The WCR assay methodology was followed with minor modifications of egg incubation for 10 days, and five plants per maize line. Mean (±SEM) NIS scores were calculated from NIS ratings of all five plants for each maize line. Comparisons between maize lines are performed in one-way ANOVA followed by multiple comparisons in a T- test at *p* = 0.05 using SAS^®^ version 9.4.

### Field root protection trial

To evaluate Vpb4Da2 transgenic maize under field conditions against high pressure WCR populations with putative field-evolved resistance, ten locations across the corn belt, where Greater Than Expected Damage (GTED) was detected on commercial *Bt* maize the previous season [34], were chosen for root protection field testing in 2017. Vpb4Da2 transgenic lines, commercial products (VT Triple PRO® with MON 88017, Herculex® RW expressing Cry34Ab1/Cry35Ab1, SmartStax®), and negative controls (commercial lepidopteran-protection maize MON 89034 expressing Cry1A.105 and Cry2Ab2, and wild-type maize) were included in the trial. The trial was set up in a completely randomized block design, consisting of three individually randomized sets of plots as three replicates at each location. Each plot contained approximately 20 plants from one maize line, planted in a 10 ft (3.04 m)-long stretch of a 2.5 ft (0.76 m)-wide row. Different plots within a row were separated by 2.5 ft (0.76 m) fallow alleys. Hybrid seeds were planted in each location in late April through mid-May. Typical agronomic practices for each location were followed and no insecticides were applied at any time. Plants were chopped at VT/T1 stages in mid to late July. Ten consecutive plants in the middle of a plot were chosen and dug. Root damage was assessed for each plant using NIS [33], and for each plot, an average NIS score was calculated from all the plants rated from the plot. Data from Colesburg, IA, and Fairbank, IA, where the highest WCR pressure (NIS ≥ 1.7 on negative controls) was observed in the trial, were selected for further analysis. Mean (±SEM) NIS scores were calculated from average NIS scores from all plots across both locations for each maize line.

### Field WCR beetle emergence trial

Wild-type maize and two Vpb4Da2 transgenic lines, selected to represent relatively high (Vpb4Da2-1) and low (Vpb4Da2-11) root expression levels, were planted in six replicates in late April to early May 2017 at two GTED locations (Leigh, Nebraska, and Shelby, Nebraska). Typical agronomic practices for each area were followed and no insecticides were applied at any time. Leaf tissue was collected from each plant at about the V2 stage and tested using PCR for transgenes. Plants that did not test as expected were manually removed, and remaining plants were thinned to approximately the same density (targeting at least 60 plants per tent). Tents were erected using 6’ x 6’ x 6’ (1.83 m x 1.83 m x 1.83 m) cage frames (Part# 1406S, BioQuip Products, Rancho Dominguez, CA) and 6’ x 6’ x 6’ (1.83 m x 1.83 m x 1.83 m) mesh screens (Part# 1406B, BioQuip Products, Rancho Dominguez, CA) over plants for each maize line prior to the onset of beetle emergence. Beetles in each tent were collected every five-seven days until no beetles were observed to emerge for 10 days. WCR beetles were identified and counted for each collection. For each location, an average beetle emergence per plant (**±**SEM) was determined for each maize line, calculated by dividing the total number of WCR beetles by the total number of plants across all six tents per maize line at this location. Percent reduction in beetle emergence was determined as the ratio of the average beetle emergence per plant for the transgenic line over the average beetle emergence per plant from the wild-type maize at the same location, as described previously [34].

### Assessing cross-resistance of Vpb4Da2 in Cry3Bb1-, DvSnf3 RNA-, and Cry34Ab1/Cry35Ab1-resistant WCR

A growth chamber whole-plant root protection assay was conducted, following the WCR assay methodology described above, to assess potential cross-resistance in Cry3Bb1- and DvSnf7 RNA-resistant WCR colonies. Wild-type maize, commercial product VT Triple PRO® expressing Cry3Bb1, transgenic maize expressing DvSnf7 RNA (ZM_S295399) [35], and a Vpb4Da2 transgenic maize line (Vpb4Da2-2) were included in the assay to test against non-diapausing susceptible lab colony (Waterman, IL), Cry3Bb1-resistant [26], or DvSnf7 RNA-resistant [35] WCR colonies.

A larval recovery assay was implemented to assess cross-resistance in Cry34Ab1/Cry35Ab1-resistant WCR. A single kernel of maize hybrid seed from a Vpb4Da2-expressing line (Vpb4Da2-2), Herculex® RW, or wild-type maize was planted into 2.36 L containers (U.S. Plastics, Lima, OH) filled with Berger BM6 potting mix. Each pot had a hole of ~6.35 mm in diameter with a 25 mm X 25 mm piece of 530 Micron Amber Lumite mesh screen (BioQuip Products, Rancho Dominguez, CA) glued over to facilitate proper drainage. For each maize line, ten plants were maintained in a growth chamber under conditions for growing maize (25°C, 16:8 h light:dark, 50% RH), and watered and fertilized (Peter’s 20-20-20 fertilizer) as necessary. Each container was infested with 30 neonates from non-diapausing susceptible (Crop Characteristics, Inc., Farmington, NM) or Cry34Ab1/Cry35Ab1-resistant WCR [36, 37] when plants reached approximately the V4 growth stage. Larvae were left to feed for 10 days, after which roots and soil were removed from pots and placed on a Berlese funnel for three days. Larvae were collected into 50% ethanol, with counts and instars recorded following collection. Individual larvae were given a numerical value based on mortality (0) or instar (1, 2, 3). Adopted from a method described previously [38], a Larval Instar Score (LIS) was used to quantify the average larval development of the 30 neonate larvae infested on each maize plant, calculated as (0*# of mortality + 1*# of 1st instar + 2*# of 2nd instar + 3*# of 3rd instar)/30. For each maize line, the mean (±SEM) LIS was calculated on LIS scores from all ten plants of the maize line. Comparisons between treatments was performed with an ANOVA analysis at *p* = 0.05 for each WCR population, based on observed LIS across all treatments using the statistical model y_ijk_ = μ+T_i_+C_j_+(TC)_ij_+e_ijk_ where μ is the overall mean across all combination of maize lines and colonies in all replicates, T_i_ for diet treatment effect from maize line i, Cj for rootworm colony effect from colony j, (TC)_ij_ for interaction effect specific between maize line i and colony j, and e_ijk_ for the residual effect for replication k. A square root transformation was applied to account for the count nature of the data. All analyses were conducted using SAS^®^ version 9.4.

## Results

### Discovery of Vpb4Da2, a new *Bt* insecticidal protein with highly selective activity against WCR

Upon inspecting the whole genome assembly of *Bt* strain EG6657, a putative β-pore forming protein with 937 amino acids (GenBank accession number AZJ95709) was identified based on sequence homology to members of the bacterial exotoxin-B β-PFP family [39, 40]. Compared to *Bt* insecticidal proteins used in current transgenic crops, it has a distinct domain architecture consisting of a PA14 Pfam domain followed by Binary_toxB, Binary_toxB_2, and Binary_toxB_3 domains. Using a high-throughput screening pipeline based on diet-overlay bioassays at a discriminating concentration of 31.25 μg/cm^2^, this protein was found to cause potent growth inhibition and high mortality against WCR larvae but not against NCR (Table 1). The protein with confirmed WCR activity (data not shown) was further tested in diet-incorporation bioassays against a panel of additional insects from Coleoptera, Lepidoptera, and Hemiptera at 500 μg/g diet. No impacts were observed against any of the neonates tested in these assays (Table 2), indicating that this new WCR-active protein is highly selective in its insecticidal activity spectrum.

**Table 1:** Vpb4Da2 is highly active against Western corn rootworm but not Northern corn rootworm.

Tested Insect	# Insects Tested	% Survival (Average ±SD)		Stunting Score (Average ±SD)	
		Buffer Control	Vpb4Da2 (31.25 μg/cm^2^)	Buffer Control	Vpb4Da2 (31.25 μg/cm^2^)
WCR	32	93.3 (±7.8)	24.4 (±12.4)	0.0 (±0.0)	3.0 (±0.0)
NCR	24	100.0 (±0.0)	95.2 (±8.3)	0.0 (±0.0)	0.0 (±0.0)

Average survival and stunting scores (±SD) of insect larvae are shown in five-day diet-overlay bioassays with Vpb4Da2 at 31.25 μg/cm^2^.

**Table 2:** Survival and average body mass of insect neonates in diet-overlay bioassays with Vpb4Da2 at 500 μg/g.

Tested Insect	Species	# Insects Tested	% Survival			Average Body Mass (mg)		
			Water Control	Buffer Control	Vpb4Da2 (500 μg/g)	Water Control	Buffer Control	Vpb4Da2 (500 μg/g)
SCR	*Diabrotica undecimpunctata*	36	93.3	91.7	94.4	2.6	2.3	2.5
CPB	*Leptinotarsa decemlineata*	24	100.0	100.0	100.0	7.3	7.7	7.2
MBB	*Epilachna varivestis*	32	100.0	100.0	100.0	1.4	1.6	1.7
CEW	*Helicoverpa zea*	16	100.0	100.0	100.0	133.7	124.8	125.5
ECB	*Ostrinia nubilalis*	24	100.0	100.0	100.0	17.9	17.1	17.1
FAW	*Spodoptera frugiperda*	16	100.0	100.0	93.8	156.8	174.9	143.3
WTPB	*Lygus hesperus*	32	93.3	91.7	94.4	2.6	2.3	2.5

SCR: Southern corn rootworm; CPB: Colorado potato beetle; MBB: Mexican bean beetle; CEW: Corn earworm; ECB: European corn borer; FAW: Fall armyworm; WTPB: Western tarnished plant bug.

The closest homolog to this new protein at the time of its discovery was Vip4Aa1 (GenBank accession number AEB52299.1; Reassigned as Vpb4Aa1), a protein with no reported insecticidal activity [15, 40]. Alignment between the two protein sequences demonstrates an overall amino acid sequence identity of 57% (sequence similarity of 72%). The sequence of this protein was submitted to the *Bacillus thuringiensis* Toxin Nomenclature, initially assigned as Vip4Da2 and recently renamed as Vpb4Da2 as a member of the Vpb4 (formally Vip4) family [3, 4].

### Vpb4Da2 provides commercial-level root protection and WCR control in transgenic maize

**Fig 1:**
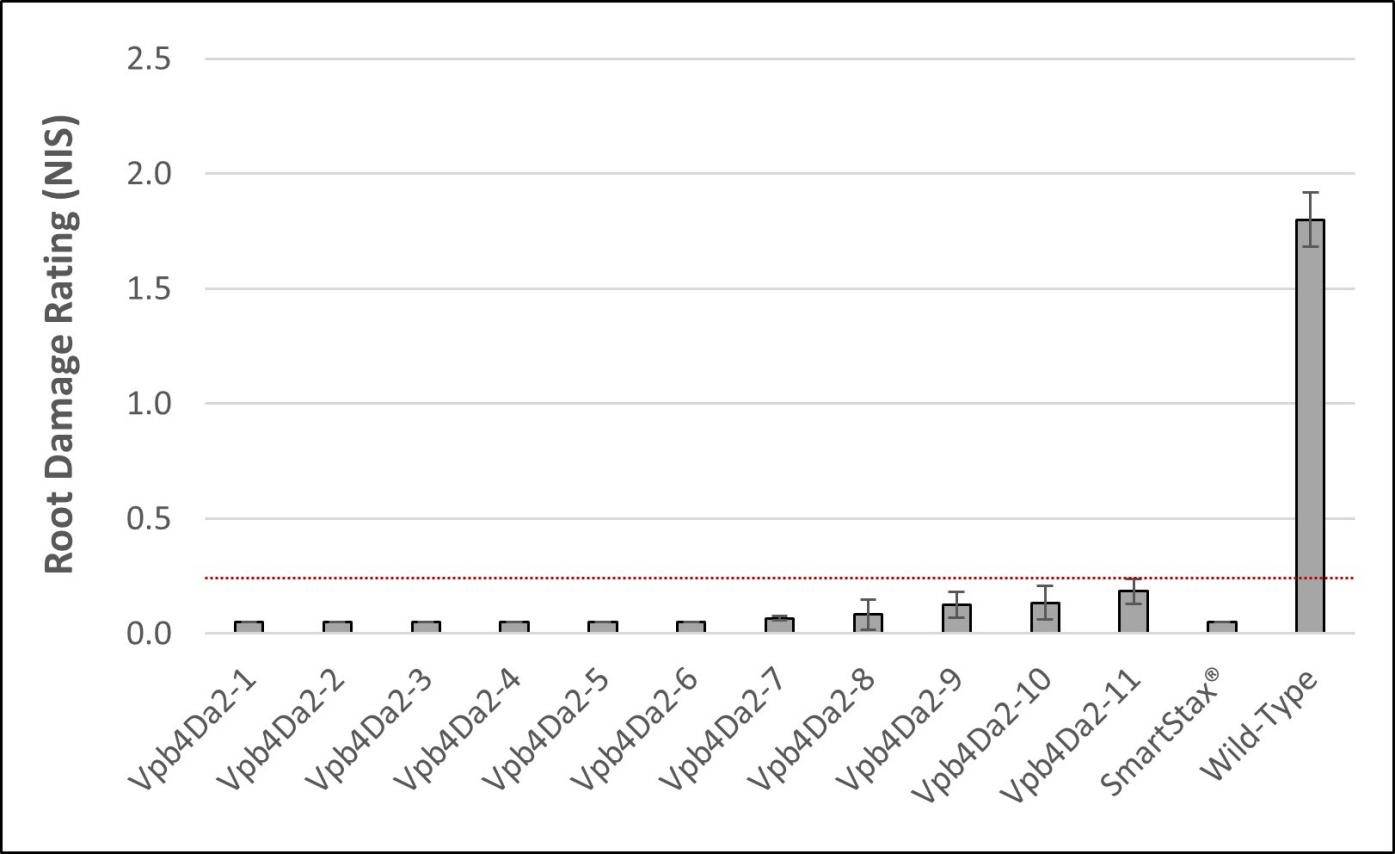
Vpb4Da2 transgenic maize provides commercial-level root protection against WCR in a growth chamber whole-plant assay. Mean (±SEM) NIS in growth chamber whole-plant root protection assays are shown, in which approximately 2,000 eggs from a lab WCR colony (Waterman) were infested on individual maize plants from Vpb4Da2 transgenic lines, SmartStax® expressing both Cry3Bb1 and Cry34Ab1/Cry35Ab1, and wild-type maize control. Dotted line in red color shows the commercial threshold of NIS 0.25, error bars represent SEM.

A synthetic sequence encoding the full-length Vpb4Da2 (GenBank accession number MT611519) was designed for optimal expression in maize. It was synthesized and built into *Agrobacterium*-mediated plant transformation vectors with selected expression elements (S1 Table). Transgenic maize lines carrying a single-copy genomic insertion and stable expression of Vpb4Da2 were generated. Commercial-level root protection (at or below the NIS 0.25 threshold) was observed on all Vpb4Da2-expressing maize lines when they were evaluated in a growth chamber 24 days after infestation with approximately 2,000 WCR eggs per plant (Fig 1). Consistent with the observation that Vpb4Da2 is not active against NCR in diet bioassays, no significant (*p* = 0.05) root protection was detected on Vpb4Da2 transgenic maize against NCR when compared to the wild-type maize in a growth chamber whole-plant assay (S1 Fig). No abnormal phenotypes were observed in any transgenic maize plants expressing Vpb4Da2.

**Fig 2:**
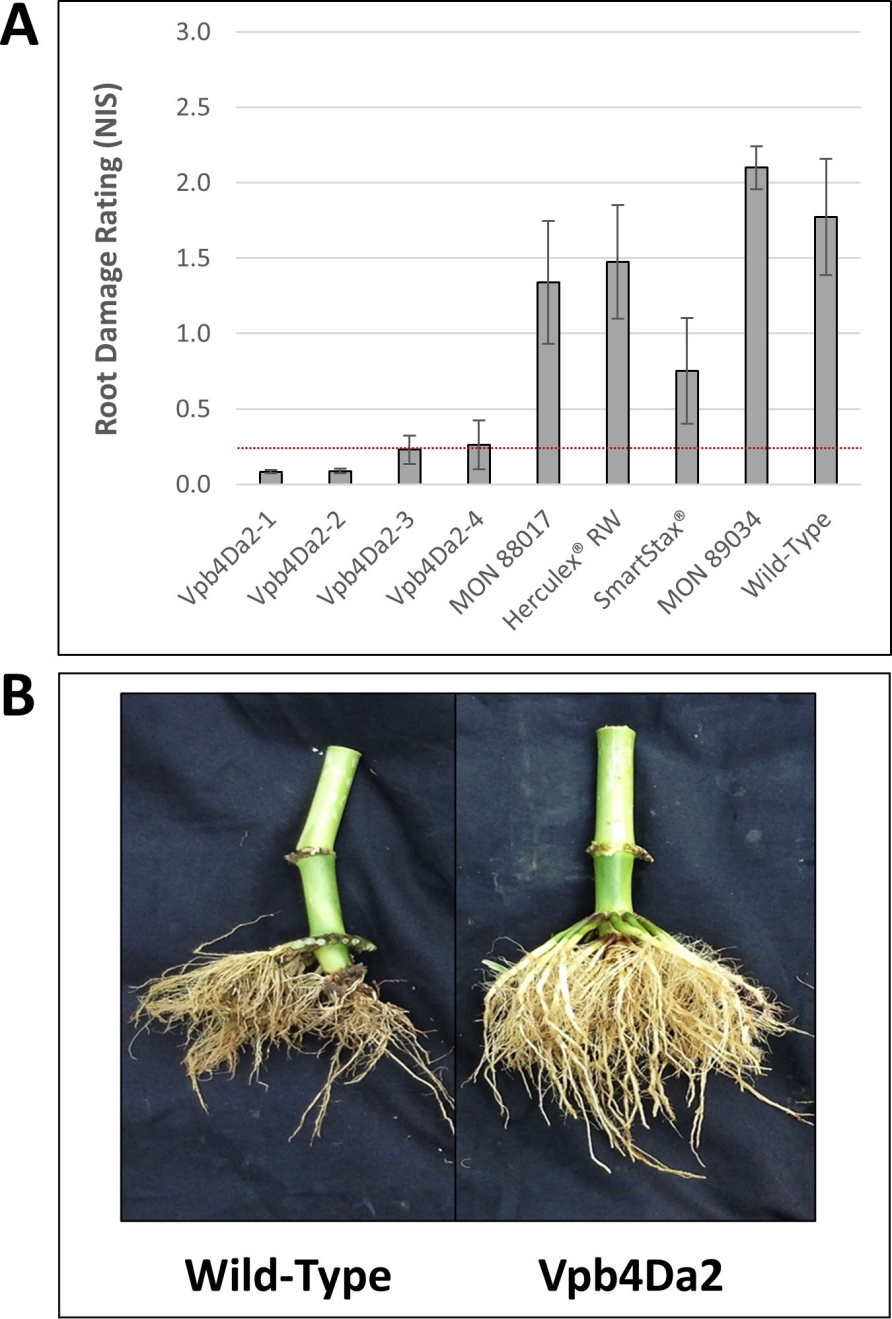
Commercial-level root protection provided by Vpb4Da2 transgenic maize against WCR under field conditions. (**A**) Mean (±SEM) NIS scores are shown across two locations with NIS ≥ 1.7 on negative controls including wild-type maize (Colesburg, IA, and Fairbank, IA) in 2017 field root protection trials. Maize lines in the trial were Vpb4Da2-expressing maize, commercial transgenic products including VT Triple PRO® carrying MON 88017, Herculex® RW, and SmartStax®, and negative controls including the commercial lepidopteran-protection trait MON 89034 and wild-type maize. Dotted line in red color shows the commercial threshold of NIS 0.25, error bars represent SEM. (**B**) Examples of roots from Vpb4Da2 transgenic maize and the wild-type maize control from Shelby, NE in 2017 field root protection trials.

To investigate the performance under field conditions against high pressure WCR populations containing putative field-evolved resistance to current commercial *Bt* traits, Vpb4Da2 transgenic maize lines were further evaluated in select GTED fields [34] in 2017. Two locations that had larval root damage NIS ≥ 1.7 on the wild-type maize or the maize line carrying the lepidopteran-protection transgenic trait MON 89034 were selected for data analyses (Fig 2A). At these two locations, substantial root damage (NIS 0.75–1.5) was also detected on commercially available *Bt* traits including MON 88017 expressing Cry3Bb1 in VT Triple PRO®, DAS-59122-7 expressing Cry34Ab1/Cry35Ab1 in Herculex® RW, and the pyramided MON 88017 × DAS-59122-7 in SmartStax® (Fig 2A). In contrast, all four Vpb4Da2-expressing transgenic lines were well protected (Fig 2A; Fig 2B), exhibiting root damage at or below the commercial threshold of NIS 0.25. These data demonstrate that Vpb4Da2 transgenic maize delivers commercial-level field protection against high-pressure WCR field populations that cause damage on transgenic maize expressing Cry3Bb1 or Cry34Ab1/Cry35Ab1.

Vpb4Da2-expressing maize lines were also investigated for their ability to reduce WCR beetle emergence in two GTED fields in 2017. An extremely high number of WCR beetles (~50 or higher per plant) emerged in tents planted with wild-type maize (Table 3). In contrast, ~2 or less WCR beetles per plant were recovered from tents planted with Vpb4Da2 transgenic maize lines, resulting in a percent reduction in adult emergence of 97.2% to 99.6% across both locations (Table 3). These results indicate that Vpb4Da2 transgenic maize provides a similar level of WCR control under field conditions as reported for transgenic maize products commercially available [34].

**Table 3:** Vpb4Da2 transgenic maize reduces WCR beetle emergence by 97% to 99%.

Maize Line	Site	# Total Plants	# Total WCR Beetles	# WCR/Plant (Mean ±SEM)	% Reduction in WCR Beetle Emergence
Vpb4Da2-1	Leigh	359	73	0.20 (±0.04)	99.6%
	Shelby	336	105	0.31 (±0.05)	99.6%
Vpb4Da2-11	Leigh	339	347	1.02 (±0.22)	97.9%
	Shelby	310	644	2.08 (±0.36)	97.2%
Wild-Type	Leigh	360	17,660	49.06 (±3.88)	-
	Shelby	336	24,915	74.15 (±3.41)	-

Emergence of WCR beetles in tents planted with Vpb4Da2 transgenic maize lines and wild-type control maize in 2017 field at Leigh, NE, and Shelby, NE. Two Vpb4Da2 transgenic lines represent the protein root expression at high (Vpb4Da2-1) and low (Vpb2Da2-11) levels, respectively.

### WCR colonies resistant to currently registered or next-generation WCR-protection traits are not cross-resistant to Vpb4Da2

To further investigate the field observation that Vpb4Da2-expressing maize were protected against the WCR populations that caused substantial damage on maize expressing Cry3Bb1 or Cry34Ab1/Cry35Ab1 suggesting possible WCR resistance, a series of studies were conducted to evaluate Vpb4Da2 against field-derived WCR colonies resistant to Cry3Bb1, Cry34Ab1/Cry35Ab1, as well as DvSnf7 RNA.

**Fig 3:**
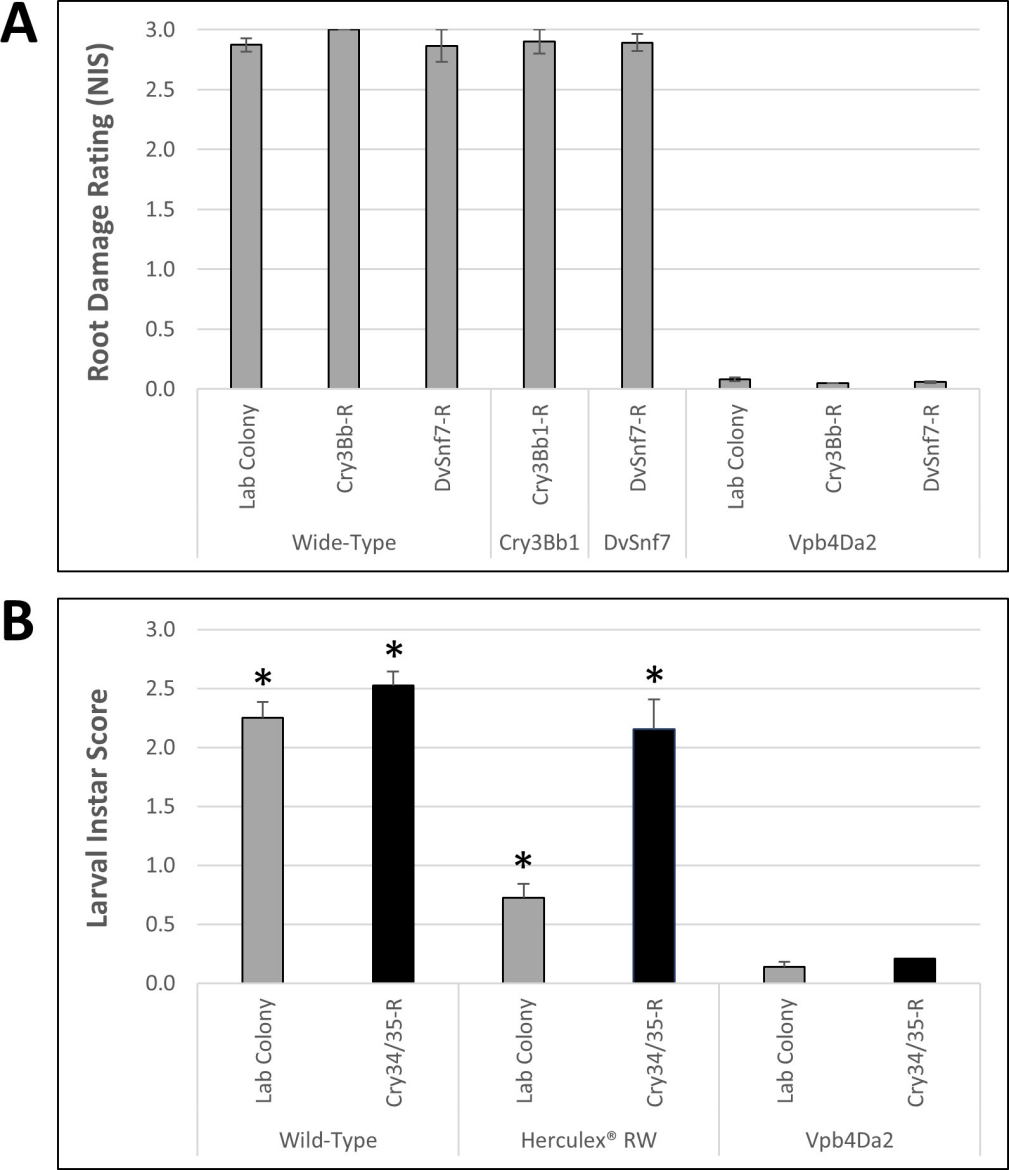
WCR colonies resistant to Cry3Bb1, DvSnf7 RNA, or Cry34Ab1/Cry35Ab1 are not cross-resistant to Vpb4Da2. (**A**) Cry3Bb1- or DvSnf7 RNA-resistant WCR colonies are not cross-resistant to Vpb4Da2. Mean (±SEM) of NIS scores in growth chamber whole-plant root protection assays are shown, in which 2,000 eggs from a susceptible lab colony (Waterman), Cry3Bb1-resistant colony, or DvSnf7 RNA-resistant colony were infested on individual maize plants from wide-type or transgenic lines expressing Vpb4Da2 (Vpb4Da2-2), Cry3Bb1, and DvSnf7 RNA, respectively. (**B**) Cry34Ab1/Cry35Ab1-resistant WCR colony is not cross-resistant to Vpb4Da2. Mean (±SEM) LIS scores are shown for susceptible (Crop Characteristics) or Cry34Ab1/Cry35Ab1-resistant WCR larvae recovered after 10 days on individual maize plants from wild-type or Herculex® RW expressing Cry34Ab1/Cry35Ab1. * LIS scores for two WCR colonies are significantly different on a same maize line at *p* = 0.05, error bars indicate SEM.

In preliminary diet-overlay bioassays, high levels of insecticidal activity were observed when Vpb4Da2 was fed to WCR neonates from the lab susceptible or Cry3Bb1-resistant colonies (data not shown). In subsequent growth chamber whole-plant root protection assays, wild-type maize sustained extreme root damage (NIS 2.9–3.0) by both Cry3Bb1-resistant and DvSnf7 RNA-resistant colonies (Fig 3A). As expected, Cry3Bb1 transgenic maize or DvSnf7 RNA-expressing line was also heavily damaged (NIS ~2.8) by Cry3Bb1-resistant or DvSnf7 RNA-resistant colonies (Fig 3A). In contrast, little root damage (NIS 0.05–0.08) was observed on Vpb4Da2 transgenic maize by either Cry3Bb1-resistant or DvSnf7 RNA-resistant colonies (Fig 3A), indicating that WCR colonies resistant to Cry3Bb1 or DvSnf7 RNA are not cross-resistant to Vpb4Da2.

To evaluate if Cry34Ab1/Cry35Ab1-resistant WCR has cross-resistance to Vpb4Da2, a ten-day larval recovery assay was implemented in which WCR larval development was quantified as the Larval Instar Score (LIS) in the scale of 0–3. Larvae from susceptible and Cry34Ab1/Cry35Ab1-resistant colonies were recovered with LIS of 2.1 and 2.5, respectively, when feeding on wild-type maize (Fig 3B). The slightly higher and statistically significant (*p* = 0.05) mean LIS score for the resistant colony on wild-type maize suggests a slightly higher fitness in this resistant population. When feeding on Cry34Ab1/Cry35Ab1-expressing Herculex® RW, a substantial increase in mortality and delay in development was observed for the control susceptible colony with LIS ~0.8 but not for the resistant WCR (LIS at ~2.2), confirming the high level of resistance in this colony to Cry34Ab1/Cry35Ab1 (Fig 3B). In comparison, Cry34Ab1/Cry35Ab1-resistant WCR sustained substantial mortality and developmental delay with LIS 0.4 on Vpb4Da2-expressing maize (Fig 3B). In addition, feeding on Vpb4Da2-expressing maize caused the lowest level of LIS (0.3–0.4) in the assay for both the susceptible and Cry34Ab1/Cry35Ab1-resistant WCR (Fig 3B). Consistent with the field observation, this study suggests that Cry34Ab1/Cry35Ab1-resistant WCR is not cross-resistant to Vpb4Da2.

## Discussion

With the evolution of WCR resistance to currently available *Bt* traits [21–24], identification of new WCR-active insecticidal proteins and other compounds has become critical [41]. In this report, we describe the discovery of a new *Bt* protein, Vpb4Da2, a new member of the Vpb4 (Vip4) insecticidal protein family with WCR activity and sequence and domain architecture distinct from other WCR-active proteins used in commercial transgenic maize.

In diet-incorporation bioassays at a relatively high concentration (500 ug/g diet), no Vpb4Da2 activity was detected against a diverse panel of insects from orders of Lepidoptera, Hemiptera, and Coleoptera (other than WCR). Unlike known WCR-active proteins in the Cry3 family and Cry34Ab1/Cry35Ab1, both of which are active against additional coleopteran pests including NCR [42, 43], Vpb4Da2 exhibited no activity against tested coleopteran insects from other genera (*Epilachna varivestis*, *Leptinotarsa decemlineata*) or even the same genus as WCR (NCR and *D*. *undecimpunctata*). The unexpected lack of activity on NCR was confirmed in a growth chamber whole-plant assay, which demonstrated no significant (*p* = 0.05) NCR root protection in the Vpb4Da2 transgenic lines that provided commercial-level root protection when tested against WCR earlier. These observations indicate that Vpb4Da2 has a highly selective insecticidal activity against WCR, making it a good candidate for developing a new tool to combat this devastating maize pest.

As a first step in developing a new WCR-protection transgenic maize, transgenic lines expressing Vpb4Da2 were generated and tested for root protection against WCR. In both growth chamber whole-plant assays where each plant was challenged with 2,000 WCR eggs, and in field tests at locations exhibiting high WCR pressure (NIS ≥ 1.7), Vpb4Da2 demonstrated commercial-level root protection against WCR, similar to current commercial *Bt* maize when they were first introduced (2003 for Cry3Bb1, 2006 for Cry34Ab1/Cry35Ab1) [42]. In addition, based on data in published reports on next-generation WCR traits, Vpb4Da2-expressing maize appears to provide at least a comparable level of root protection compared to the transgenic maize expressing DvSnf7 RNA [34] or the *Pseudomonas chlororaphis* protein, IPD072Aa [27].

Given the demonstrated ability of WCR to evolve resistance to a range of insect control technologies, the risk of resistance development to Vpb4Da2, when deployed alone or in combination with other approaches, needs to be understood. One primary determinant of resistance risk (or potential durability) for an insect protection transgenic trait is the dose level, which can be measured as the reduction in WCR beetle emergence between feeding on transgenic and wild-type maize. For example, current transgenic maize expressing Cry3Bb1 or Cry34Ab1/Cry35Ab1 caused at least 95% reduction in WCR beetle emergence when they were commercially introduced [34]. When evaluated in two fields with historically high WCR damage and suspected WCR resistance to Cry3Bb1 and Cry34Ab1/Cry35Ab1, Vpb4Da2-expressing maize resulted in a 97.2% to 99.6% reduction in WCR beetle emergence across both locations. These results suggest that Vpb4Da2-expressing maize is as good as commercial products expressing Cry3Bb1 or Cry34Ab1/Cry35Ab1 in controlling WCR.

With the evolution of resistance to current *Bt* traits targeting WCR, a key priority in developing Vpb4Da2 is to ensure that WCR populations resistant to current *Bt* proteins will not be cross-resistant to Vpb4Da2. Cross-resistance evaluations rely on a weight-of-evidence approach that is typically based on assessment of sequence diversity followed by protein binding studies, on the assumption that reduction or alteration in receptor binding is indicative of resistance [5]. However, direct testing on insect populations displaying resistance is always preferred whenever possible [26, 44]. Our results from trials in locations under GTED conditions demonstrated that Vpb4Da2 remained efficacious where Cry3Bb1 and Cry34Ab1/Cry35Ab1 resistance was suspected. In addition, we conducted growth chamber experiments in which Vpb4Da2 transgenic maize was tested against field-derived WCR colonies with confirmed resistance to Cry3Bb1, Cry34Ab1/Cry35Ab1, or DvSnf7 RNA. In all tests, Vpb4Da2 provided similar root protection or caused similar toxicity against these resistant WCR colonies as against susceptible WCR, indicating that WCR populations resistant to Cry3Bb1, Cry34Ab1/Cry35Ab1, or DvSnf7 RNA are not cross-resistant to Vpb4Da2. Because of the observed partial to full cross-resistance between Cry3Bb1 and mCry3A and eCry3.1Ab [24], it is reasonable to expect no cross-resistance by Vpb4Da2 to either mCry3A or eCry3.1Ab (yet to be determined).

With the demonstration of commercial-level root protection in Vpb4Da2 transgenic maize under field conditions, high levels of reduction in WCR beetle emergence, and absence of cross-resistance of Vpb4Da2 in Cry3Bb1-, Cry34Ab1/Cry35Ab1-, or DvSnf7 RNA-resistant colonies, the new *Bt* protein Vpb4Da2 (Vip4Da2) provides a selective, non-chemical new tool for protection against WCR.

## Supporting information

S1 Fig Vpb4Da2 transgenic maize does not provide root protection against NCR in growth chamber whole-plant assay. Mean (±SEM) NIS scores in a growth chamber whole-plant root protection assay are shown, in which 2,000 eggs from a lab NCR colony (Crop Characteristics) were infested on individual maize plants from commercial traits MON 88017 expressing Cry3Bb1, SmartStax® expressing both Cry3Bb1 and Cry34Ab1/Cry35Ab1, four Vpb4Da2 transgenic lines that are highly efficacious against WCR, and wild-type maize control. Error bars represent SEM. *Statistically different from the wild-type control using one-way ANOVA followed by multiple comparisons between each maize line and the wild-type control in T-test at the level of *p* = 0.05.
(DOCX)

S1 Table Selection of Vpb4Da2 expression cassette for ideal expression pattern and highest root protection against WCR. Leaf/root Vpb4Da2 protein expression measured by ELISA, and root damages rated in NIS in a WCR growth chamber whole-plant root protection assay are shown for Vpb4Da2 transgenic maize plants produced with three sets of expression cassettes.
(DOCX)

S1 Method Selection of plant expression cassette and Vpb4Da2 transgenic maize lines. (DOCX)
