# Supplementary material for: A new Bacillus thuringiensis protein for Western corn rootworm control
Source: PLoS One. 2020 Nov 30;15(11):e0242791. doi: 10.1371/journal.pone.0242791 (PMC7703998; doi:10.1371/journal.pone.0242791)

**S1 Fig. Vpb4Da2 transgenic maize does not provide root protection against NCR in growth chamber whole-plant assay.** Mean (±SEM) NIS scores in a growth chamber whole-plant root protection assay are shown, in which 2,000 eggs from a lab NCR colony (Crop Characteristics) were infested on individual maize plants from commercial traits MON 88017 expressing Cry3Bb1, SmartStax® expressing both Cry3Bb1 and Cry34Ab1/Cry35Ab1, four Vpb4Da2 transgenic lines that are highly efficacious against WCR, and wild-type maize control. Error bars represent SEM. *Statistically different from the wild-type control using one-way ANOVA followed by multiple comparisons between each maize line and the wild-type control in T-test at the level of *p*=0.05.


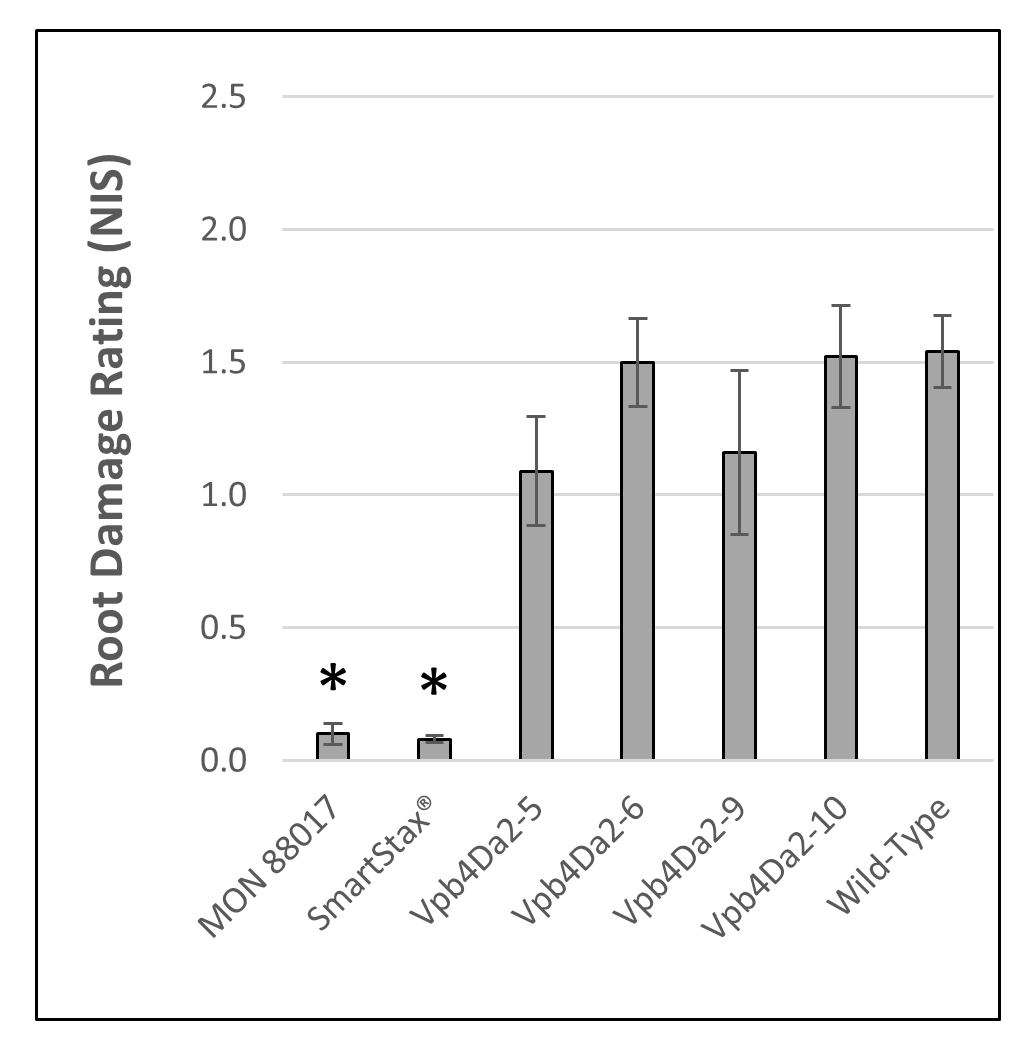

Supplement: S1 Fig — Mean (±SEM) NIS scores in a growth chamber whole-plant root protection assay are shown, in which 2,000 eggs from a lab NCR colony (Crop Characteristics) were infested on individual maize plants from commercial traits MON 88017 expressing Cry3Bb1, SmartStax® expressing both Cry3Bb1 and Cry34Ab1/Cry35Ab1, four Vpb4Da2 transgenic lines that are highly efficacious against WCR, and wild-type maize control. Error bars represent SEM. *Statistically different from the wild-type control using one-way ANOVA followed by multiple comparisons between each maize line and the wild-type control in T-test at the level of p = 0.05. (DOCX) [file pone.0242791.s001.docx]
