# Supplementary material for: A new Bacillus thuringiensis protein for Western corn rootworm control
Source: PLoS One. 2020 Nov 30;15(11):e0242791. doi: 10.1371/journal.pone.0242791 (PMC7703998; doi:10.1371/journal.pone.0242791)
Supplement: S1 Table — Leaf/root Vpb4Da2 protein expression measured by ELISA, and root damages rated in NIS in a WCR growth chamber whole-plant root protection assay are shown for Vpb4Da2 transgenic maize plants produced with three sets of expression cassettes. (DOCX) [file pone.0242791.s002.docx]

**S1 Table. Selection of Vpb4Da2 expression cassette for ideal expression pattern and highest root protection against WCR.** Leaf/root Vpb4Da2 protein expression measured by ELISA, and root damages rated in NIS in a WCR growth chamber whole-plant root protection assay are shown for Vpb4Da2 transgenic maize plants produced with three sets of expression cassettes.

| Expression Cassette | Enhancer/Promoter/5'-Leader/Intron | 3'-UTR | Root Expression (µg/g dry weight ±SEM) | Leaf Expression (µg/g dry weight ±SEM) | Root Damage by WCR  (NIS ±SEM) |
| --- | --- | --- | --- | --- | --- |
| C1 | E-DaMVr +P/L-Zm.Ltp +I-SETit.Act4 | T-SETit.Ams1 | 45.4 ±19.2 | 20.8 ±12.7 | 0.18 ±0.15 |
| C2 | E-DaMV +P/L-SETit.Ifr +I-SETit.eIF5A3-2 | T-Cl.Hsp16.9 | 64.5 ±19.7 | 95.7 ±42.8 | 0.54 ±0.31 |
| C3 | P/L/I-Zm.UbqM1 | T-Os.Ltp | 36.0 ±12.9 | 126.7 ±90.5 | 0.63 ±0.35 |
| SmartStax® | | | | | 0.07 ±0.03 |
| Wild-Type | | | | | 2.0 ±0.22 |
