# Supplementary material for: A new Bacillus thuringiensis protein for Western corn rootworm control
Source: PLoS One. 2020 Nov 30;15(11):e0242791. doi: 10.1371/journal.pone.0242791 (PMC7703998; doi:10.1371/journal.pone.0242791)
Supplement: S1 Method — (DOCX) [file pone.0242791.s003.docx]

**S1 Method. Selection of plant expression cassette and Vpb4Da2 transgenic maize lines**

Multiple expression cassettes were constructed and screened for optimal expression of Vpb4Da2 and efficacy of root protection against WCR (Table S1). Cassette C1 includes a recombinant enhancer E-DaMVr (GenBank accession number MT611512) derived from sequences from Dahlia mosaic virus Holland isolate (GenBank accession number EU090957.1) and Dahlia mosaic virus-p2 (GenBank accession number JX272320), the promoter (P-Zm.Ltp; 1,201 bp upstream of the transcription start site) and 5’ leader (L-Zm.Ltp; 93 bp downstream) of the gene encoding maize lipid transfer protein Ltp (GenBank accession number MT611508), the first intron (I-SETit.Act4) of *Setaria italica* actin 4 gene (GenBank accession number MT611515), and the 3' UTR (T-SETit.Ams1) from *Setaria italica* S-adenosylmethionine synthase 1 gene (GenBank accession number MT611517). Cassette C2 consists of an enhancer (E-DaMV.Flt) from Dahlia mosaic virus (GenBank accession number MT611512 ), the promoter (P-SETit.Ifr) and 5’ leader (L-SETit.Ifr) from *Setaria italica* isoflavone reductase gene (GenBank accession number MT611520), the second intron (I-SETit.eIF5A3-2) from *Setaria italica*  eukaryotic initiation factor 5A gene (GenBank accession number MT611521), and the 3’ UTR (T-Cl.Hsp16.9) derived from the gene encoding *Coix lacryma-jobi* heat shock protein 16.9 (GenBank accession number MT611516). Cassette C3 contains the promoter, 5’ leader, and first intron (P/L/I-Zm.UbqM1) from *Zea mays cv. Mexicana* polyubiquitin gene M1 (GenBank accession number MH931399.1; Nucleotide 1-2,008), and a 3’ UTR (T-Os.Ltp) from *Oryza sativa* lipid transfer protein gene (GenBank MH931404.1; Nucleotide 1-300).

Transgenic maize lines generated from the three cassettes demonstrated different expression levels of Vpb4Da2 protein in root and leaf tissues, as well as different levels of root protection against WCR (S1 Table). Among the three, the cassette C1 with the E-DaMVr enhanced Zm.LTP promoter/leader and SETit.Act4 intron provided the best, commercial-level of root protection against WCR in growth chamber whole-plant root protection assays as demonstrated by the commercial transgenic trait SmartStax®. In addition, this cassette also demonstrated the lowest level of expression of Vpb4Da2 in non-target leaf tissue among the three. Selected Vpb4Da2-expressing maize lines from this construct were thus advanced to field testing and other studies.
